# Incidence, prevalence, and global burden of myocarditis among individuals aged 65 and older from 1990 to 2021 across 204 countries: a critical re-analysis of data from the global burden of disease study

**DOI:** 10.3389/fcvm.2026.1611616

**Published:** 2026-03-03

**Authors:** Ang Li, Jingyu Tan, Jing Hu, Yongyi Bai

**Affiliations:** 1 Department of Cardiology, Second Medical Center, Chinese PLA General Hospital, Beijing, China; 2 Institute of Gastroenterology of PLA, Southwest Hospital, Army Medical University, Chongqing, China

**Keywords:** elderly, global burden, incidence, myocarditis, prevalance

## Abstract

Myocarditis is an inflammatory disease of the heart muscle that can lead to significant morbidity and mortality, particularly in the elderly population (≥65 years). This study aimed to provide a comprehensive global, regional, and national burden analysis of myocarditis from 1990 to 2021, with a prediction for 2050. We employed a variety of methodologies, including the estimation of age-standardized incidence rates, prevalence, disability-adjusted life years (DALYs), and their estimated annual percentage change (EAPC). Additionally, we analyzed age, sex, and temporal trends using line graphs and trend charts, revealing the shifts in disease burden across different demographics. Joinpoint regression analysis was performed to identify significant changes in burden over time, while the relationship between disease burden and the Socio-Demographic Index (SDI) was explored through a curve plot with a dot plot overlay. Age-period-cohort analysis was conducted to assess the influences of age effects, period effects, and cohort effects on disease burden. Decomposition analysis was employed to understand the impacts of population growth, aging, and epidemiological changes on the overall burden. Furthermore, we identified key risk factors contributing to myocarditis burden through curves and attributable risk ratios. Finally, Bayesian Age-Period-Cohort (BAPC) modeling was utilized to project the disease burden of myocarditis globally until 2050. Evidence suggests that a pivotal shift in disease burden occurred after 2010, with low temperature emerging as a primary risk factor for mortality. Our findings indicate a concerning trend in the increasing burden of myocarditis among the aging population, underscoring the urgent need for targeted public health strategies and further research to mitigate this growing health crisis.

## 1 Introduction

Myocarditis, an inflammatory disease of the myocardium, has emerged as a significant public health concern, particularly among the elderly population aged 65 years and above. The global burden of myocarditis has been increasingly recognized, with studies indicating varying incidence rates and associated mortality across different regions and demographics. This condition not only leads to considerable morbidity but also poses challenges in terms of diagnosis and management, especially in older adults who often present with atypical symptoms and comorbidities. The complexity of myocarditis, influenced by factors such as infectious agents, autoimmune responses, and environmental exposures, necessitates a comprehensive understanding of its epidemiological trends and risk factors.

Recent literature has highlighted the rising incidence of myocarditis, particularly during the COVID-19 pandemic, where viral infections have been linked to increased myocarditis cases in the elderly (1). Additionally, a significant body of research has focused on the disability-adjusted life years (DALYs) associated with myocarditis, underscoring its impact on healthcare systems and the need for effective intervention strategies (2). The Global Burden of Disease (GBD) studies have provided valuable data on the age-standardized rates of myocarditis, revealing substantial regional disparities and trends over time (3). The Global Burden of Disease (GBD) study uses measurements such as incidence, deaths, YLL, and disability-adjusted life years (DALYs), serving as a platform for researchers and health workers around the world to obtain a comprehensive understanding of a particular disease. To date, there are only a few recent GBD studies that comprehensively discuss the situation of myocarditis. Consequently, more detailed studies and analyses are necessary, which may help policymakers allocate and optimize the limited medical resources to reduce the burden of this disease.

While the existing literature offers insights into the burden of myocarditis, notable gaps remain in understanding the age, sex, and time-related variations in disease incidence and outcomes. For instance, the literature indicates that age-specific incidence rates show an upward trend, particularly among older adults, but detailed analyses are often limited to specific geographic regions or demographic groups (4). Furthermore, the relationship between socio-demographic indices (SDI) and myocarditis burden has not been thoroughly explored, highlighting the need for targeted research to inform public health policies (2).

To address these gaps, our study employs a robust methodology to analyze the burden of myocarditis from 1990 to 2021, focusing on the elderly population. By utilizing data from the GBD and conducting a joinpoint regression analysis, we aim to identify significant trends in incidence, prevalence, and mortality rates across various global regions. The analysis period of this study encompassed the initial phase of the COVID-19 pandemic. With an extended temporal coverage from 1990 to 2021, enhanced age stratification into seven subgroups, and a comprehensive assessment of ambient temperature as a modifiable risk factor, this study offers robust epidemiological evidence on the evolving burden of myocarditis in older populations during a critical public health crisis. This study also seeks to explore the association between myocarditis burden and socio-demographic factors, thus contributing to a more nuanced understanding of the disease's epidemiology.

Ultimately, the findings from this research will serve as a critical foundation for developing targeted interventions and policies aimed at reducing the burden of myocarditis among the aging population. Given the expected rise in the elderly demographic globally, addressing the health implications of myocarditis is imperative to enhance patient outcomes and optimize healthcare resources (1–3).

## 2 Methods

### 2.1 Data sources

All the data for this study were derived from the GBD 2021 database (https://vizhub.healthdata.org/gbd-results/). This database encompasses disease burden data for 371 diseases and 88 risk factors, covering 204 countries and regions globally, and employs a unified approach for data collection and indicator evaluation (5). The study concentrated on extracting data of elderly myocarditis patients aged 65 and above during the period from 1990 to 2021, in order to assess the prevalence trend and disease burden. The extracted data include the following dimensions: demographic characteristics such as age group (65+), gender, year (1990–2021), geographical division (204 countries/regions worldwide, encompassing World Health Organization member states, 5 SDI regions, and 21 GBD regions), and socio-demographic index (SDI): a socioeconomic background indicator for measuring regional health status, integrating the total fertility rate of the population under 25 years old, the average education level of the population aged 15 and above, and per capita lagged disposable income. The 204 countries/regions were classified into five development levels based on SDI: low, lower-middle, middle, upper-middle, and high. Regional classification: The 21 GBD regions were divided based on epidemiological similarities and geographical proximity.

### 2.2 Analytical indicators

This study utilized the following indicators to describe the disease burden of myocarditis in the elderly aged 65 and above: estimated values of incidence rate, prevalence rate, and DALYs (Disability-Adjusted Life Years) and their 95% uncertainty intervals (UI). Age-standardized incidence rate, age-standardized prevalence rate, and age-standardized DALYs rate were calculated based on the age structure of the GBD world standard population.

### 2.3 Statistical methods

Excel 2021 and R language version 4.3.3 were adopted to analyze the disease burden data of myocarditis in the elderly worldwide from 1990 to 2021. After cleaning and organizing the saved data, statistical analysis and graphing were conducted. A significance level of *P* < 0.05 was considered statistically significant.

#### 2.3.1 Age group analysis

In accordance with the age group principle of GBD 2021, the study subjects were divided into the following 7 age groups: 65–69 years old, 70–74 years old, 75–79 years old, 80–84 years old, 85–89 years old, 90–94 years old, and 95+ years old, to explore the dynamic changes in the disease burden of myocarditis in different age groups and genders.

#### 2.3.2 Trend analysis

Percentage change (EAPC): R software was employed to calculate and visualize the percentage change in the disease burden of myocarditis for each age, gender, region, and country from 1990 to 2021. The trend of age-standardized rates was quantified by the Estimated Annual Percentage Change (EAPC). An EAPC and its 95% confidence interval lower limit > 0 indicated an upward trend in the age-standardized rate; conversely, a lower limit < 0 indicated a downward trend (6). Joinpoint Regression Model: The Joinpoint regression model was utilized to analyze the temporal trends from 1990 to 2021, calculating the Average Annual Percent Change (AAPC) and the Annual Percent Change (APC) for each time period. In this study, we analyzed the trend changes in the incidence of myocarditis. Given that the dependent variable follows a Poisson distribution, we finally selected the log—linear model. The grid search method (GSM) was employed to determine the optimal number of join points, aiming to identify the time points at which the disease burden significantly altered. When the 95% confidence interval included 0, the trend was considered stable. An AAPC or APC significantly greater than or less than 0 indicated an upward or downward trend in the relevant indicator, respectively (7). Model fitting and AAPC/APC calculation were accomplished through the Joinpoint software (version 4.9.0.0), and visualization was performed using R language.

#### 2.3.3 Correlation analysis

Pearson correlation analysis was implemented to assess the correlation between SDI and the age-standardized rate of myocarditis. Simultaneously, the proportion of elderly myocarditis deaths related to risk factors such as low and high temperatures was analyzed.

### 2.4 Decomposition and prediction analysis

#### 2.4.1 Decomposition analysis

Decomposition methods were applied to quantify the independent contributions of population growth, aging, and epidemiological changes to the disease burden of myocarditis while keeping other factors fixed (8).

#### 2.4.2 Age-Period-Cohort analysis

The Age-Period-Cohort (APC) model was used to evaluate the dynamic changes in the disease affected by age, period, and cohort factors. The APC model is based on the Poisson distribution and decomposes variables from the three dimensions of age, period, and cohort to analyze their influence on the risk of myocarditis incidence or death (9).

#### 2.4.3 Bayesian age-period-cohort model (BAPC)

On the basis of the APC model, the Bayesian Markov Chain Monte Carlo algorithm was incorporated to reduce the difficulty of parameter estimation caused by the linear relationship among the three factors. The model was used to predict the disease burden of myocarditis from 2022 to 2050 (10). The Bayesian Age-Period-Cohort (BAPC) modeling process consists of three key components: specification of prior distributions, formulation of the likelihood function, and estimation of effects through posterior sampling. In this study, a first-order random walk prior was applied to model temporal dependencies; the likelihood function was defined as the joint probability of all observed data, expressed as the product of Poisson distributions across age groups and time periods; posterior inference was performed using the Markov Chain Monte Carlo (MCMC) algorithm. Age groups were categorized in 5-year intervals: 65–69, 70–74, 75–79, 80–84, 85–89, 90–94, and 95+, totaling seven groups. The analysis spanned 17 calendar years from 1990 to 2021, while birth cohorts were grouped into 18 five-year intervals (1895–1955). Relative risk (RR) estimates were derived, and their 95% credible intervals (CrI) were obtained by computing the percentiles of the posterior distribution. Initially, an Age-Period-Cohort (APC) model was fitted using the Epi package in R to examine the temporal trends in myocarditis incidence and mortality across regions. Models incorporating different combinations of age, period, and cohort effects were compared based on residual deviance to identify the optimal random walk (RW) specification. Subsequently, the BAPC package was employed to construct the final BAPC model and project global myocarditis incidence, prevalence, and DALYs from 2021 to 2050.

## 3 Results

### 3.1 Global burden of myocarditis in the elderly

In 2021, the age-standardized incidence rate of myocarditis among the elderly was 56.06 (95% UI: 31.74, 87.31) per 100,000 people, the age-standardized prevalence rate was 17.44 (95% UI: 11.14, 25.75) per 100,000 people, and the age-standardized DALYs was 36.43 (95% UI: 27.54, 43.93) per 100,000 person-years. This implies that there were 418,372.20 (95% UI: 236,761.12, 650,424.86) new cases of myocarditis in 2021, among which 217,360.40 (95% UI: 123,022.50, 337,506.01) were men and 201,011.80 (95% UI: 113,621.65, 313,146.14) were women. In 2021, there were 129,437.77 (95% UI: 82,487.07, 191,244.49) existing cases of myocarditis, including 64,466.24 (95% UI: 40,475.25, 96,052.39) men and 64,971.53 (95% UI: 42,005.79, 95,567.17) women. The DALYs attributed to myocarditis in 2021 were 264,233.76 (95% UI: 200,048.34, 318,656.91) person-years, including 131,879.11 (95% UI: 93,616.91, 170,602.51) person-years for men and 132,354.65 (95% UI: 93,995.29, 168,521.32) person-years for women. From 1990 to 2021, the EAPC of the age-standardized incidence rate of myocarditis in the elderly was −0.21129, the EAPC of the age-standardized prevalence rate was 0.03907, and the EAPC of the age-standardized DALYs was −1.03571, indicating that the disease burden of myocarditis among the elderly has generally shown a downward trend worldwide (See Table 1 for details).

**Table 1:** Age-standardized myocarditis burden results for the global population, five SDI regions, and 21 GBD regions.

Location	Incidence			Prevalence			DALYs		
	1990 (per 100,000 population, 95% UI)	2021 (per 100,000 population, 95% UI)	EAPCs (95% CI)	1990 (per 100,000 population, 95% UI)	2021 (per 100,000 population, 95% UI)	EAPCs (95% CI)	1990 (per 100,000 population, 95% UI)	2021 (per 100,000 population, 95% UI)	EAPCs (95% CI)
Global	57.26 (32.07, 90.01)	56.06 (31.74, 87.31)	−0.21 (0.50, 0.08)	16.43 (10.18, 24.83)	17.44 (11.14, 25.75)	0.04 (−0.19, 0.27)	45.23 (35.55, 55.42)	36.43 (27.54, 43.93)	−1.04 (−1.38, −0.69)
SDI									
High SDI	60.48 (33.79, 95.24)	58.13 (33.47, 89.74)	−0.47 (−0.83, −0.12)	15.86 (9.24, 24.78)	16.34 (10.19, 24.51)	−0.17 (−0.48, 0.14)	12.57 (10.86, 14.24)	11.41 (9.67, 12.88)	−0.57 (−0.95, −0.19)
High-middle SDI	56.56 (31.59, 89.37)	54.18 (30.56, 84.73)	−0.27 (−0.57, 0.03)	18.64 (12.10, 27.11)	19.19 (12.43, 27.89)	−0.17 (−0.52, 0.18)	98.37 (79.24, 115.45)	53.73 (42.84, 64.09)	−2.68 (−3.19, −2.18)
Middle SDI	56.63 (31.70, 88.93)	57.51 (32.20, 90.16)	0.05 (−0.19, 0.30)	17.08 (10.80, 25.44)	19.42 (12.58, 28.41)	0.43 (0.25, 0.61)	57.52 (39.53, 77.19)	59.23 (37.13, 79.27)	0.09 (−0.22, 0.41)
Low-middle SDI	54.27 (30.52, 84.84)	53.95 (30.33, 84.27)	0.00 (−0.24, 0.24)	14.29 (8.35, 22.21)	14.41 (8.47, 22.35)	0.04 (−0.16, 0.23)	28.88 (13.89, 54.20)	24.12 (14.85, 41.84)	−0.44 (−0.51, −0.36)
Low SDI	52.69 (29.67, 82.55)	52.74 (29.71, 82.63)	0.01 (−0.23, 0.25)	13.45 (7.71, 21.25)	13.52 (7.75, 21.29)	0.02 (−0.16, 0.22)	27.11 (9.85, 58.30)	21.12 (9.51, 45.58)	−0.47 (−0.59, −0.35)
GBD 21 regions									
Andean Latin America	58.45 (32.57, 90.73)	57.77 (32.89, 89.01)	−0.03 (−0.32, 0.26)	15.97 (9.55, 24.48)	15.28 (9.08, 23.63)	−0.22 (−0.47, 0.03)	21.26 (10.89, 36.94)	6.31 (4.06, 9.45)	−4.37 (−4.58, −4.16)
Australasia	53.31 (29.70, 83.87)	53.93 (30.30, 84.31)	−0.04 (−0.39, 0.31)	15.50 (9.61, 23.39)	15.88 (9.88, 23.81)	−0.23 (−0.52, 0.05)	25.12 (18.66, 32.94)	9.13 (7.13, 11.52)	−3.89 (−4.24, −3.53)
Caribbean	55.93 (31.34, 87.12)	55.63 (31.18, 86.65)	−0.00 (−0.27, 0.27)	14.26 (8.26, 22.42)	14.54 (8.55, 22.72)	0.07 (−0.20, 0.35)	10.73 (7.47, 16.71)	11.98 (8.99, 16.88)	0.25 (−0.19,0.69)
Central Asia	49.39 (27.39, 77.76)	50.84 (28.25, 79.79)	0.05 (−0.33, 0.43)	13.65 (8.11, 21.01)	15.29 (9.51, 23.01)	0.48 (0.16, 0.81)	35.42 (25.96, 46.26)	63.74 (47.02, 85.88)	2.61 (2.08,3.15)
Central Europe	52.08 (29.23, 81.65)	52.15 (29.24, 81.73)	−0.10 (−0.42, 0.23)	15.93 (10.08, 23.59)	18.90 (12.47, 27.30)	0.43 (0.13, 0.72)	88.26 (65.12, 119.13)	108.62 (82.66, 138.88)	−0.48 (−0.95, −0.02)
Central Latin America	58.19 (32.48, 90.72)	57.67 (32.18, 89.89)	−0.01 (−0.28, 0.26)	14.56 (8.29, 22.97)	15.29 (8.98, 23.64)	0.22 (−0.05, 0.49)	4.97 (4.04, 5.98)	9.52 (8.06, 10.92)	2.54 (2.22,2.86)
Central Sub-Saharan Africa	50.98 (28.66, 79.98)	49.90 (28.00, 78.28)	−0.02 (−0.26, 0.21)	12.58 (7.05, 19.99)	12.33 (6.89, 19.58)	−0.03 (−0.23, 0.18)	33.46 (11.73, 81.91)	24.57 (8.03, 75.34)	−1.04 (−1.14, −0.94)
East Asia	57.10 (31.75, 90.67)	59.01 (33.13, 93.11)	0.13 (−0.15, 0.41)	19.40 (12.54, 28.44)	23.57 (15.40, 33.90)	0.71 (0.52,0.90)	83.71 (58.72, 115.29)	87.82 (56.29, 117.80)	0.23 (−0.24, 0.69)
Eastern Europe	51.74 (29.16, 80.97)	52.32 (29.49, 81.85)	0.01 (−0.22, 0.23)	16.05 (10.06, 23.81)	14.81 (8.96, 22.58)	−0.24 (−0.39, −0.09)	45.48 (39.51, 51.31)	33.29 (27.31, 40.35)	−0.90 (−1.25, −0.56)
Eastern Sub-Saharan Africa	52.67 (29.67, 82.57)	52.48 (29.58, 82.28)	0.01 (−0.23, 0.25)	13.27 (7.53, 21.04)	13.20 (7.48, 20.97)	−0.01 (−0.19, 0.17)	8.81 (3.56, 19.49)	5.32 (2.08, 13.27)	−1.72 (−1.97, −1.47)
High-income Asia Pacific	59.82 (33.62, 94.00)	57.95 (32.65, 90.83)	−0.28 (−0.69, 0.13)	15.19 (8.74, 23.83)	16.09 (9.84, 24.59)	−0.10 (−0.44, 0.25)	12.46 (9.93, 16.14)	10.24 (8.35, 12.08)	−1.36 (−1.76, −0.96)
High-income North America	69.00 (38.10, 109.43)	59.69 (35.88, 88.55)	−1.16 (−1.46, −0.85)	17.46 (9.86, 27.73)	15.80 (9.86, 23.59)	−0.96 (−1.24, −0.67)	7.01 (5.80, 8.15)	5.98 (5.03, 6.84)	−0.88 (−1.11, −0.66)
North Africa and Middle East	39.55 (21.84, 62.04)	39.74 (21.95, 62.28)	0.01 (−0.19, 0.22)	11.52 (7.11, 17.29)	11.76 (7.31, 17.76)	0.08 (−0.14, 0.29)	49.75 (19.47, 130.10)	33.28 (16.85, 80.48)	−1.20 (−1.45, −0.94)
Oceania	54.80 (30.38, 85.97)	54.96 (30.46, 86.20)	0.00 (−0.28, 0.29)	13.53 (7.58, 21.46)	13.56 (7.59, 21.50)	0.00 (−0.25, 0.26)	6.37 (2.46, 13.69)	4.90 (1.84, 11.61)	−0.70 (−1.94, 0.56)
South Asia	55.41 (31.22, 87.05)	54.77 (30.90, 85.98)	−0.02 (−0.25, 0.22)	14.31 (8.22, 22.41)	14.25 (8.21, 22.26)	0.00 (−0.18, 0.18)	53.66 (31.20, 84.64)	58.76 (34.22, 92.01)	−0.21 (−0.33, −0.09)
Southeast Asia	58.54 (32.88, 91.31)	58.20 (32.69, 91.04)	−0.02 (−0.33, 0.28)	16.72 (10.21, 25.41)	16.97 (10.32, 25.74)	−0.03 (−0.28, 0.22)	25.83 (11.17, 45.71)	21.65 (12.41, 35.16)	−2.19 (−2.36, −2.03)
Southern Latin America	54.05 (30.18, 84.30)	55.78 (31.89, 86.17)	0.11 (−0.16, 0.39)	14.11 (8.14, 21.90)	14.46 (8.52, 22.30)	0.09 (−0.17, 0.35)	42.98 (25.56, 76.27)	26.31 (18.26, 41.43)	−1.90 (−2.31, −1.48)
Southern Sub-Saharan Africa	53.24 (30.04, 83.36)	53.05 (29.93, 83.11)	0.01 (−0.23, 0.24)	13.24 (7.38, 21.04)	13.17 (7.32, 20.93)	−0.01 (−0.24, 0.23)	16.75 (12.23, 22.55)	9.55 (7.53, 11.98)	−2.47 (−2.87, −2.07)
Tropical Latin America	59.66 (33.06, 93.18)	59.36 (32.90, 92.72)	−0.00 (−0.27, 0.26)	15.77 (9.22, 24.54)	17.14 (10.38, 26.09)	0.23 (−0.04, 0.50)	18.60 (9.72, 31.01)	10.04 (6.97, 17.07)	−0.61 (−1.18, −0.04)
Western Europe	58.29 (32.65, 91.88)	56.09 (31.81, 87.57)	−0.31 (−0.70, 0.08)	17.35 (10.94, 25.83)	16.70 (10.39, 25.14)	−0.40 (−0.87, 0.06)	14.33 (12.24, 16.32)	14.40 (12.12, 16.47)	−5.24 (−6.03, −4.44)
Western Sub-Saharan Africa	53.05 (29.92, 83.10)	52.99 (29.87, 83.05)	−0.01 (−0.25, 0.23)	13.29 (7.48, 21.14)	13.20 (7.41, 21.03)	−0.04 (−0.22, 0.15)	61.95 (48.89, 72.95)	16.52 (12.98, 19.22)	−4.01 (−4.20, −3.81)

### 3.2 Regional burden of myocarditis in the elderly

Among the five SDI regions, the High SDI region had the highest age-standardized incidence rate of myocarditis in 2021 [58.13 (95% UI: 33.47, 89.74)], while the Middle SDI region had the highest age-standardized prevalence rate [19.42 (95% UI: 12.58, 28.41)] and age-standardized DALYs [59.23 (95% UI: 37.13, 79.27)]. The Low-middle SDI region had the lowest age-standardized incidence rate [52.74 (95% UI: 29.71, 82.63)] and age-standardized prevalence rate [13.52 (95% UI: 7.75, 21.29)], and the High SDI region had the lowest age-standardized DALYs [11.41 (95% UI: 9.67, 12.88)]. From the perspective of temporal trends, the disease burden of myocarditis in different SDI regions exhibited different trends. Specifically, the incidence (EAPC = −0.47, 95% UI: −0.83, −0.12) and prevalence (EAPC = −0.17, 95% UI: −0.48, 0.14) in the High SDI region and the DALYs (EAPC = 1.58, 95% UI: 0.64, 2.52) in the High-middle SDI region decreased most significantly. In the Middle SDI region, the incidence (EAPC = 0.05, 95% UI: −0.19, 0.30), prevalence (EAPC = 0.43, 95% UI: 0.25, 0.61), and DALYs (EAPC = 0.09, 95% UI: −0.22, 0.41) showed an increasing trend (see Table 1 for details).

Among the 21 GBD regions, the age-standardized incidence rate in High-income North America in 2021 was the highest (59.69, 95% UI: 35.88, 88.55), the age-standardized prevalence rate in East Asia was the highest (23.57, 95% UI: 15.40, 33.90), and the age-standardized DALYs in Central Europe was the highest (108.62, 95% UI: 82.66, 138.88), while the age-standardized incidence rate in North Africa and Middle East was the lowest (39.74, 95% UI: 21.95, 62.28), the age-standardized prevalence rate was the lowest (11.76, 95% UI: 7.31, 17.76), and the age-standardized DALYs in Oceania was the lowest (4.90, 95% UI: 1.84, 11.61). From the perspective of temporal trends, the disease burden in different GBD regions presented diverse trends. Among them, the incidence (EAPC = 0.13, 95% UI: −0.15, 0.41) and prevalence (EAPC = 0.71, 95% UI: 0.52, 0.90) in East Asia and the DALYs (EAPC = 2.61, 95% UI: 2.08, 3.15) in Central Asia increased significantly; the incidence (EAPC = −0.10, 95% UI: −0.42, 0.23) in Central Europe, the prevalence (EAPC = −0.40, 95% UI: −0.87, 0.06) in Western Europe, and the DALYs (EAPC = −5.24, 95% UI: −6.03, −4.44) in Western Europe decreased significantly.

### 3.3 National and regional burden of myocarditis in the elderly

**Figure 1:**
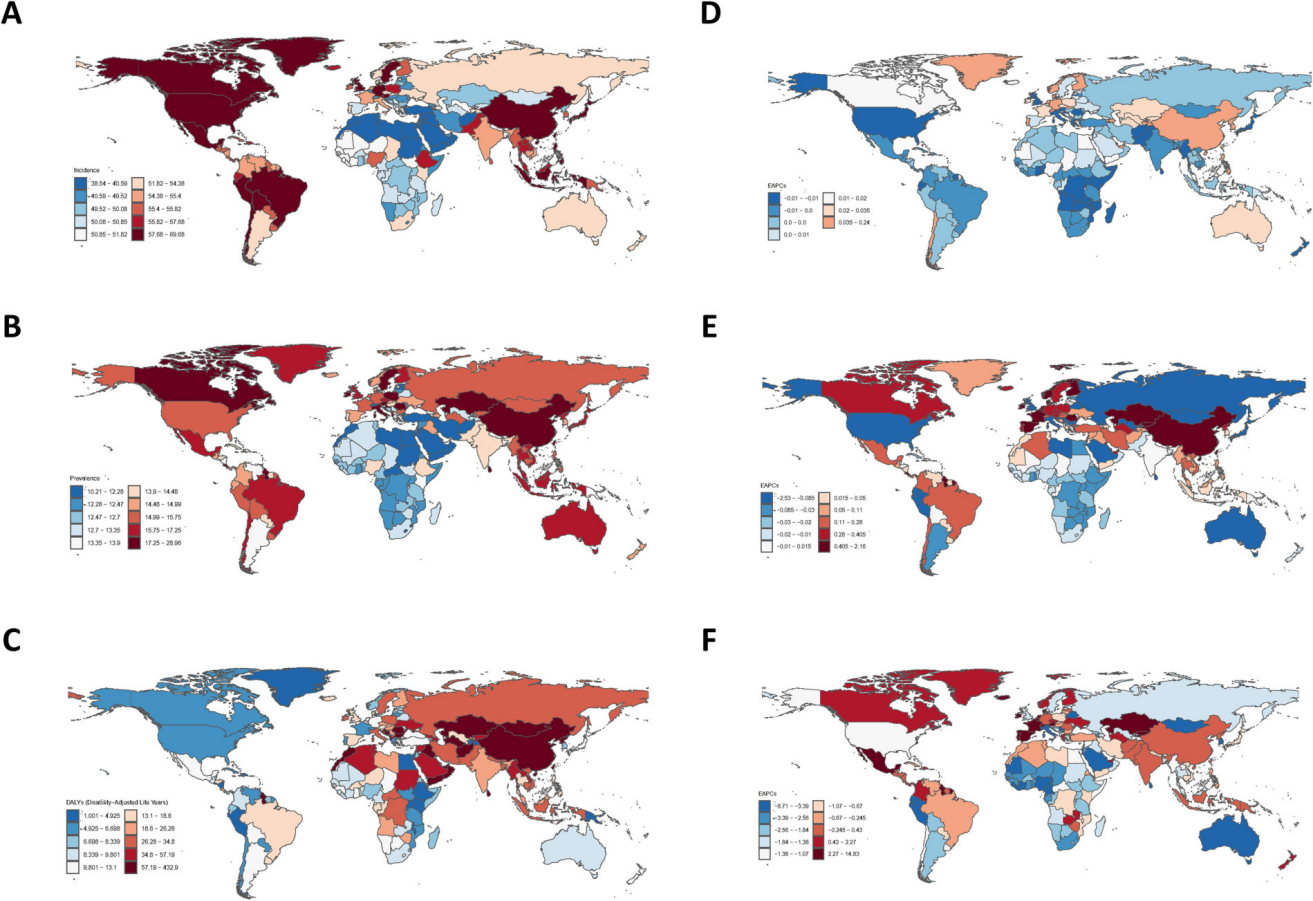
Global burden of myocarditis in 204 countries and regions in 2021 [**(A)** Age-standardized incidence rate; **(B)** Age-standardized prevalence rate; **(C)** Age-standardized DALYs; **(D)** EAPC of age-standardized incidence rate; **(E)** EAPC of age-standardized prevalence rate; **(F)** EAPC of age-standardized DALYs].

In 2021, the age-standardized incidence rate of myocarditis among the elderly was the highest in Greenland, reaching 69.68 (95% UI: 39.03, 108.91) per 100,000 people. Romania had the highest age-standardized prevalence rate, which was 28.96 (95% UI: 17.93, 43.22) per 100,000 people. Kazakhstan had the highest age-standardized DALYs, amounting to 173.29 (95% UI: 104.95, 261.40) per 100,000 person-years. Conversely, the age-standardized incidence rate of myocarditis among the elderly was the lowest in Palestine, which was 38.54 (95% UI: 21.28, 60.41) per 100,000 people. The age-standardized prevalence rate was the lowest in Jordan, which was 10.21 (95% UI: 5.87, 15.88) per 100,000 people, and the age-standardized DALYs was the lowest in Cook Islands, which was 0.62 (95% UI: 0.21, 1.27) per 100,000 person-years (see Figures 1A–C for details). Overall, the overall disease burden indicators in China were also at a relatively high level, in which the age-standardized incidence rate (EAPC = 0.14; 95% UI: −0.14, 0.42), age-standardized prevalence rate (EAPC = 0.68; 95% UI: 0.49, 0.87) and the age-standardized DALYs (EAPC = 0.24; 95% UI: −0.25, 0.73) were shown. Longitudinal time trend analysis revealed that the disease burden of myocarditis in Italy showed a distinct downward trend. Moreover, the age-standardized incidence rate in the United States of America and the age-standardized prevalence rate and DALYs in Sri Lanka also exhibited downward trends. Beyond these, the trends of the disease burden of myocarditis in the elderly in other countries and regions were not pronounced (the 95% CI of EAPC encompassed 0) (See Figures 1D–F for details). Among them, the upward trends of the age-standardized incidence rate in the United Arab Emirates (EAPC = 0.24, 95% UI: 0.06, 0.42), the prevalence rate in Kazakhstan (EAPC = 2.16, 95% UI: 1.82, 2.50), and the DALYs in Kazakhstan (EAPC = 14.83, 95% UI: 13.54, 16.14) were the most conspicuous.

### 3.4 Age-gender association analysis of myocarditis in the elderly and trends in disease burden

**Figure 2:**
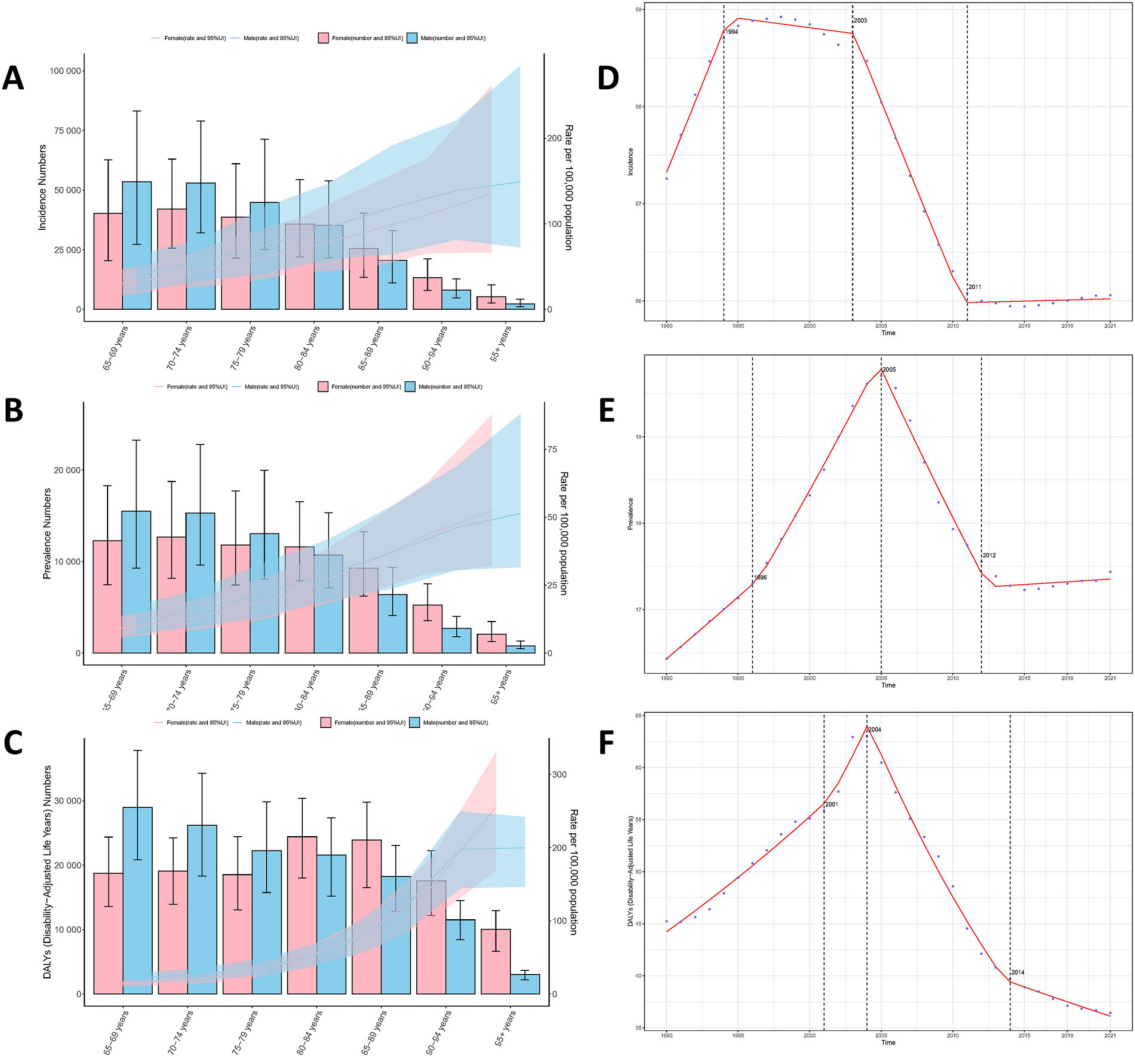
Age-gender variations in the disease burden of myocarditis and the results of joinpoint regression analysis [**(A)** incidence; **(B)** prevalence; **(C)** DALYs; **(D)** joinpoint analysis of incidence; **(E)** joinpoint analysis of prevalence; **(F)** joinpoint analysis of DALYs].

The age-gender association analysis reveals that in 2021, the incidence, prevalence, and DALYs of myocarditis among different age groups over 65 years old decreased with age, though this change was not statistically significant. Furthermore, the disease burden among elderly men was higher than that among women. For detailed information, please refer to Figures 2A–C.

Joinpoint regression analysis demonstrates that from 1990 to 2021, the incidence, prevalence, and DALYs of myocarditis in the elderly globally generally exhibited an initial upward trend followed by a downward trend. Specifically, the AAPC of incidence was 2.575 (95% CI: 2.528, 2.621), the AAPC of prevalence was 22.545 (95% CI: 21.740, 23.350), and the AAPC of DALYs was 0.654 (95% CI: 0.630, 0.678). The years when significant alterations occurred in the three disease burden indicators were mainly concentrated in 2003, 2004, and 2005. For detailed data, please consult Figures 2D–F. Since 2010, the upward trend of the disease burden of myocarditis has slightly decelerated, and DALYs have entered a downward phase.

### 3.5 Association between the disease burden of myocarditis in the elderly and SDI

**Figure 3:**
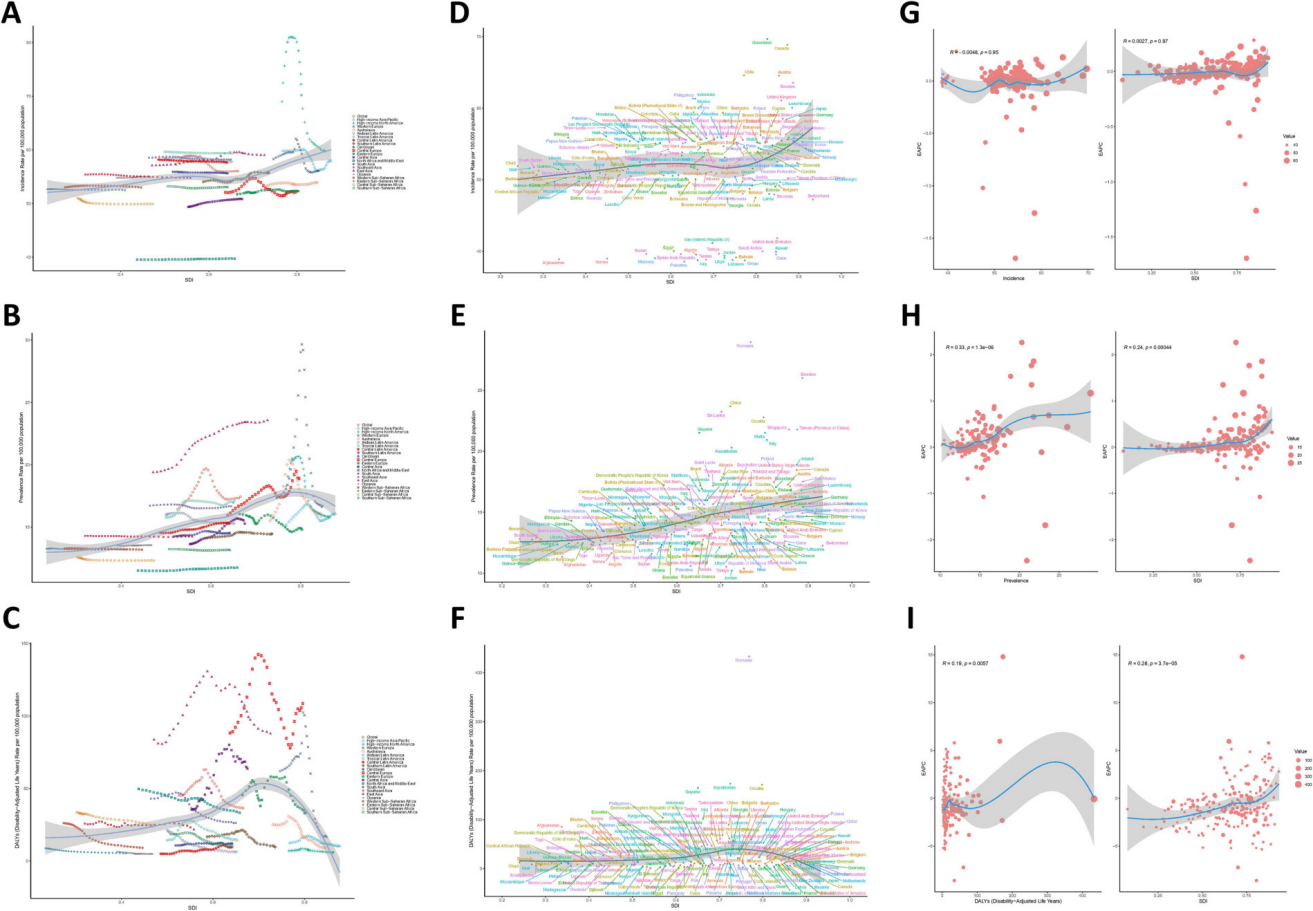
SDI analysis results [**(A)** incidence rate of 21 regions; **(B)** prevalence rate of 21 regions; **(C)** DALYs of 21 regions; **(E)** incidence rate of 204 countries; **(F)** prevalence rate of 204 countries; **(G)** DALYs of 204 countries; **(G)** EAPC of incidence rate; **(H)** EAPC of prevalence rate; **(I)** EAPC of DALYs].

On a global scale and within the 21 GBD regions, a nonlinear relationship exists between SDI and the age-standardized incidence, prevalence, and DALYs of myocarditis. Specifically, as SDI rises, the disease burden of myocarditis exhibits a gradual downward trend, reaching its minimum when SDI is approximately 0.55. Subsequently, as SDI continues to increase, the disease burden of myocarditis demonstrates a slow upward trend, and the disease burden of elderly myocarditis patients is relatively mitigated when SDI is around 0.8. Among them, the disease burden in regions such as High Income North America, Western Europe, and Central Europe has undergone significant changes among the 21 GBD regions, as depicted in Figures 3A–C. Among the 204 countries, the incidence (*p* = 2.571 × 10^−3^) and prevalence (*p* = 5.719 × 10^−13^) of myocarditis increase with the escalation of SDI, while DALYs (*p* = 2.705 × 10^−2^) do not display a significant correlation. Additionally, the EAPC of prevalence and DALYs also increases with the growth of SDI, indicating that the disease burden of myocarditis becomes more severe in countries with higher SDI. For detailed illustrations, please refer to Figures 3G–I.

### 3.6 Results of age-period-cohort analysis of the disease burden of myocarditis in the elderly

**Figure 4:**
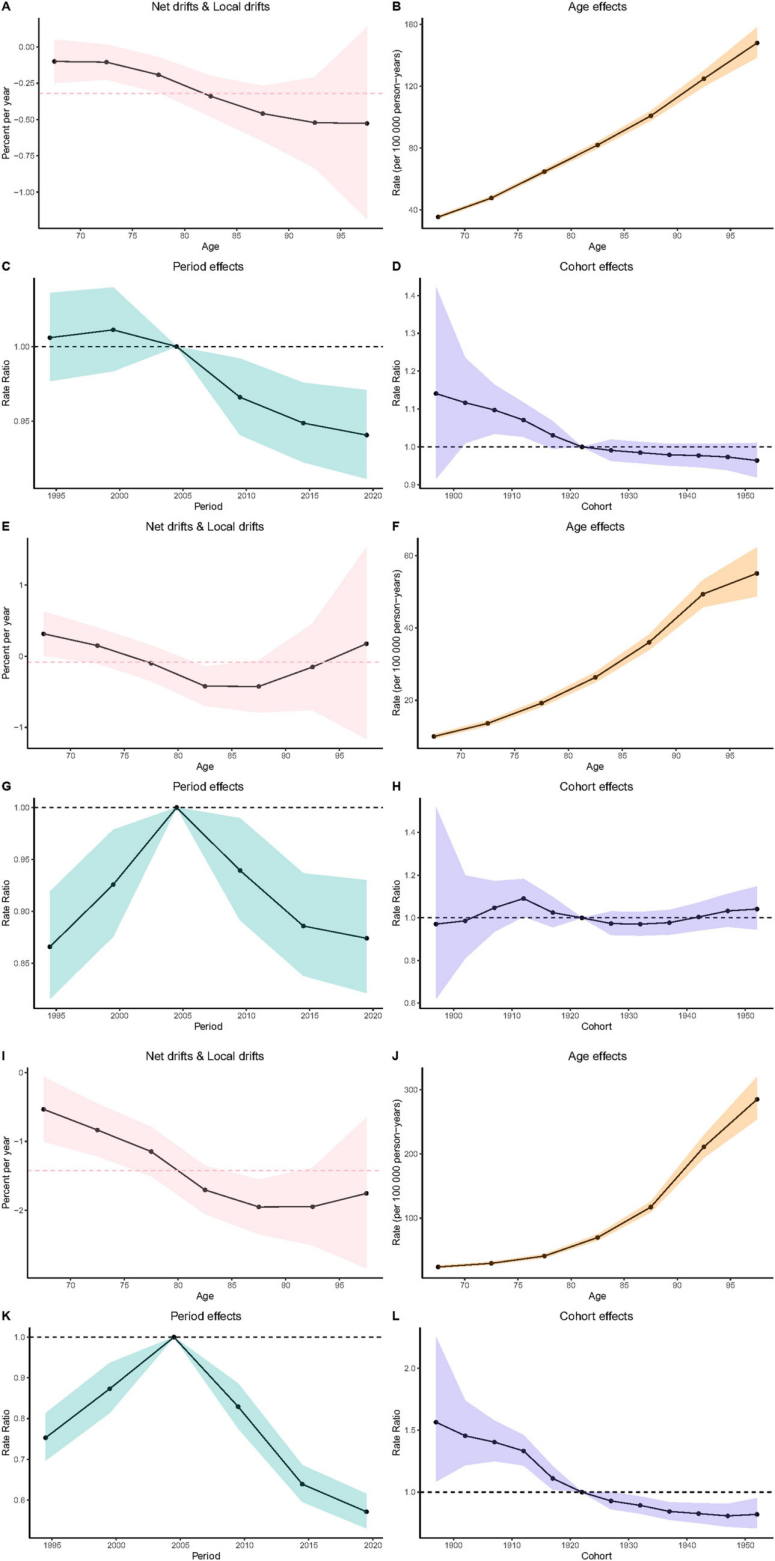
Results of age-period-cohort analysis [**(A–D)** Age-period-cohort effects on incidence; **(E–H)** Age-period-cohort effects on prevalence; **(I–L)** Age-period-cohort effects on DALYs].

The results of the age-period-cohort analysis of the incidence, prevalence, and DALYs of myocarditis in the elderly exhibit a similar pattern, that is, the proportion decreases with age in different age groups, as shown in Figures 4A,E,I. The age effect analysis indicates that the disease burden of myocarditis shows a significant upward trend with advancing age, as depicted in Figures 4B,F,J. The period effect analysis reveals that the disease burden of myocarditis gradually increases over the period from 1990 to 2021, and then declines year by year after 2005, as presented in Figures 4C,G,K. The cohort effect analysis shows that the disease burden of myocarditis in later-born cohorts (after 1922) is significantly lower than that in earlier-born cohorts (before 1922), as detailed in Figures 4D,H,L.

### 3.7 Decomposition analysis results of the disease burden of myocarditis in the elderly

**Figure 5:**
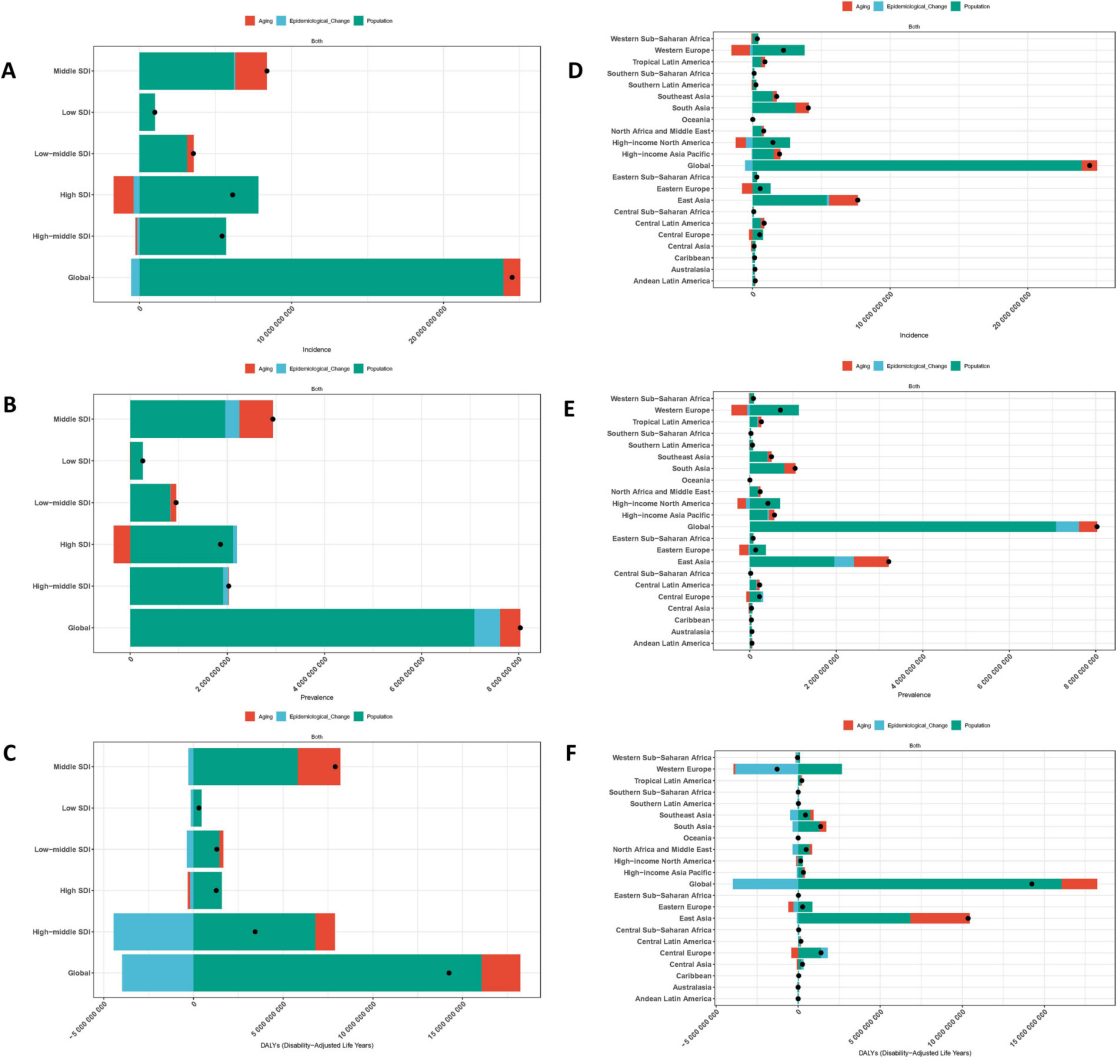
Decomposition analysis results [**(A)** incidence in the world and 5 SDI regions; **(B)** prevalence in the world and 5 SDI regions; **(C)** DALYs in the world and 5 SDI regions; **(D)** incidence in 21 GBD regions; **(E)** prevalence in 21 GBD regions; **(F)** DALYs in 21 GBD regions].

The decomposition analysis results disclose that the influences of population growth, aging, and epidemiological changes on the disease burden of myocarditis in the global population, the five SDI regions, and the 21 GBD regions present a similar contribution pattern. Specifically, except for the High SDI and High-Middle SDI regions, Western Europe, High-Income North America, and Eastern Europe, where aging significantly reduces the disease burden of myocarditis, population growth, aging, and epidemiological changes in the remaining regions all contribute to an increase in the disease burden of myocarditis. Among them, population growth is the primary cause of the aggravated disease burden of myocarditis. For detailed information, please refer to Figure 5.

### 3.8 Prediction analysis results of the disease burden of myocarditis in the elderly

**Figure 6:**
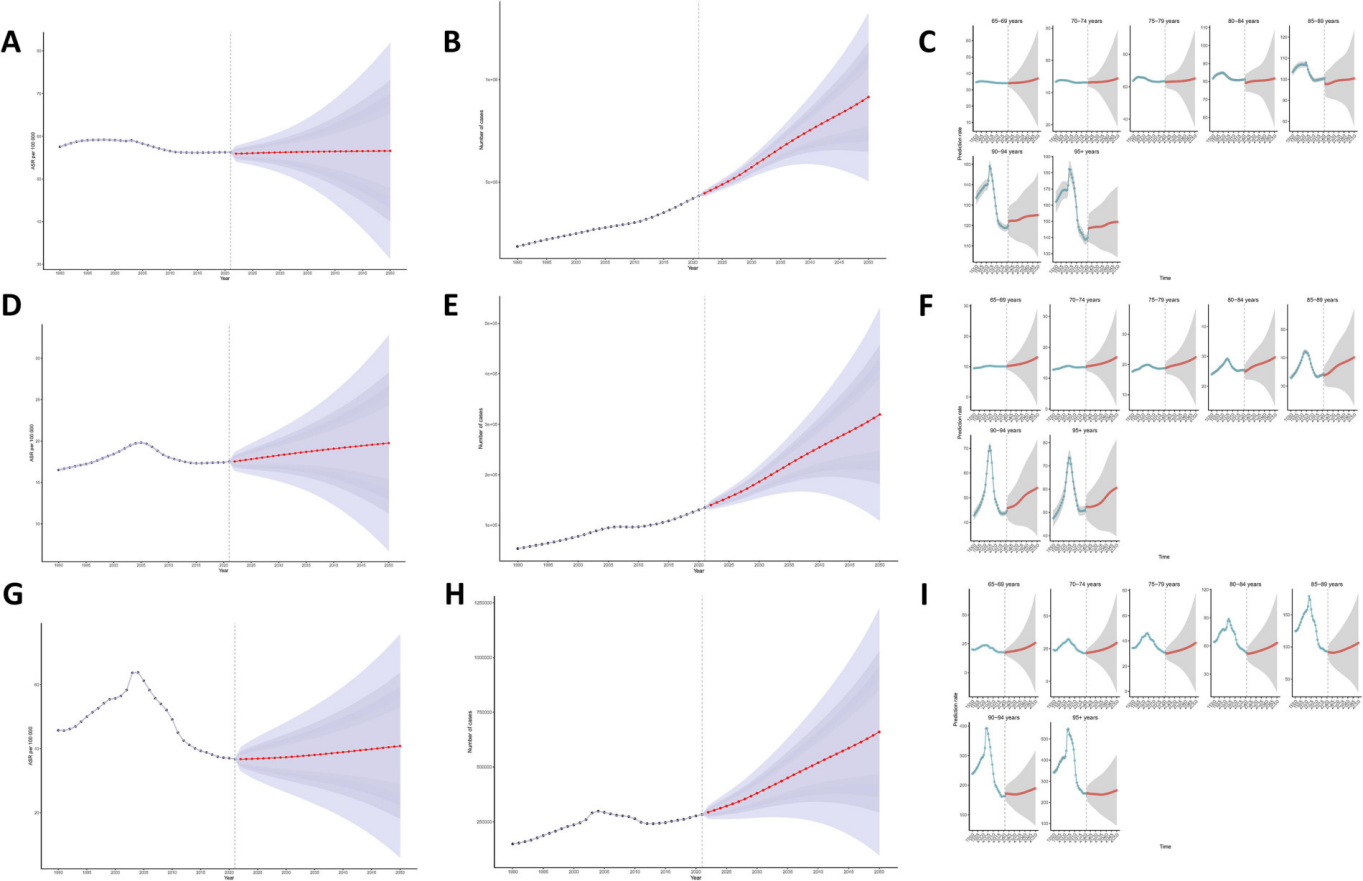
Prediction analysis results [**(A)** Age-standardized incidence rate; **(B)** actual incidence rate; **(C)** incidence rate by different age groups; **(D)** Age-standardized prevalence rate; **(E)** actual prevalence rate; **(F)** prevalence rate by different age groups; **(G)** Age-standardized DALYs; **(H)** actual DALYs; **(I)** DALYs by different age groups].

The prediction analysis results suggest that from 2022 to 2050, the disease burden of myocarditis in the elderly will further escalate globally. By 2050, the age-standardized incidence rate, prevalence rate, and DALYs of myocarditis in individuals over 65 years old will increase to 56.58 (95% CI: 31.32, 81.83) per 100,000 people, 19.75 (95% CI: 6.73, 32.77) per 100,000 people, and 40.84 (95% CI: 5.99, 75.68) per 100,000 person-years, respectively. This implies that by 2050, there will be an estimated 915,002.04 (95% CI: 96,955.37–1,223,923.20) new cases of viral myocarditis among individuals aged 65 years and older, 319,444.85 (95% CI: 108,882.20–530,007.49) prevalent cases in the same population, and a cumulative loss of 660,439.28 (95% CI: 96,955.37–1,223,923.19) life-years. Furthermore, our predictive analysis across different age groups revealed that the disease burden of myocarditis exhibited a significant upward trend in all age categories. Notably, this upward trend became more pronounced with increasing age (Figures 6A–H).

### 3.9 The impact of low and high temperature environments on the disease burden of myocarditis in the elderly

**Figure 7:**
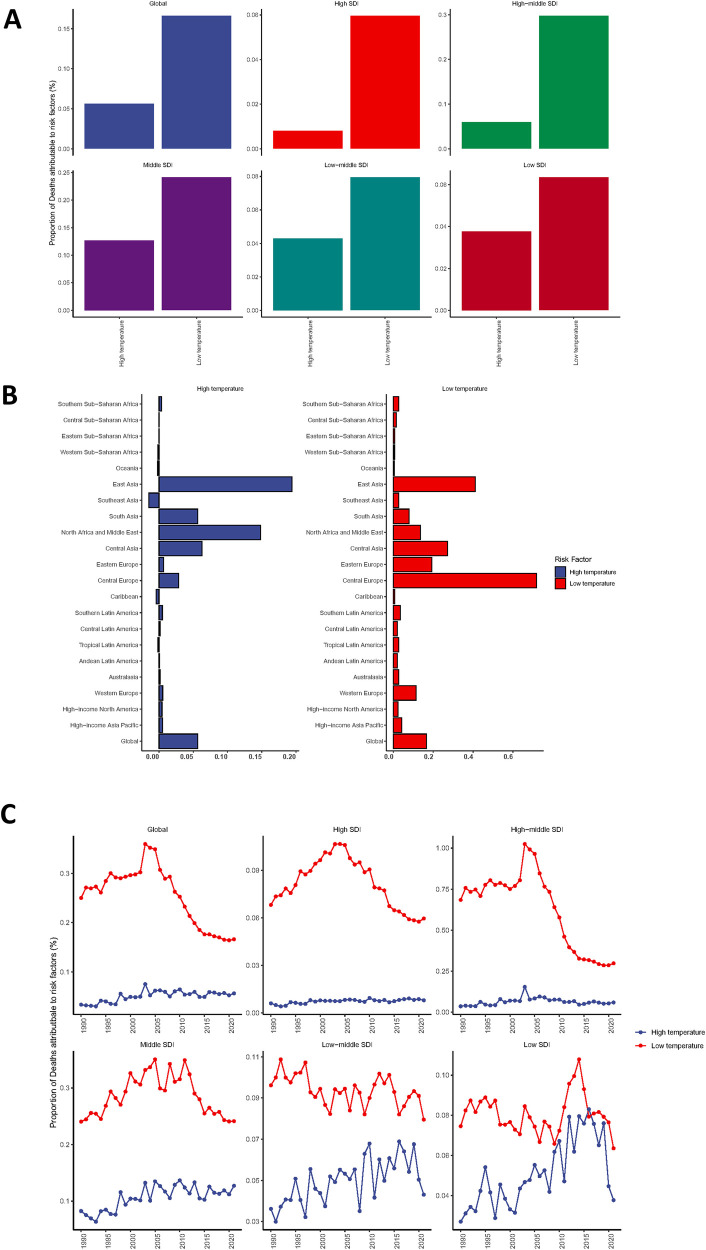
The impact of exposure to different temperature environments on elderly patients with myocarditis [**(A)** trends in global and 5 SDI region myocarditis mortality rates in 2021; **(B)** trends in myocarditis mortality rates in 21 regions in 2021; **(C)** trends in exposure to low and high temperature environments over time].

Based on existing reports on the risk factors for mortality among elderly myocarditis patients, there are two risk factors contributing to the mortality of elderly myocarditis patients, namely exposure to low and high temperatures. From a global and five SDI region perspective, exposure to low temperatures is the predominant risk factor for mortality among myocarditis patients (Figure 7A). Exposure to high temperatures is closely associated with higher mortality rates in East Asia, North Africa and the Middle East, Central Asia, and South Asia, while exposure to low temperatures may primarily affect mortality rates in Central Europe, Eastern Europe, East Asia, and Central Asia (Figure 7B). With the passage of time, the disease burden of myocarditis caused by different risk factors is on the rise, with the most significant increase observed in the disease burden resulting from exposure to low temperatures (Figure 7C).

## 4 Discussion

Myocarditis is an inflammatory disease of the myocardium that can lead to significant morbidity and mortality, particularly among the elderly population. This condition is characterized by an immune-mediated attack on heart muscle tissue, which can result in a spectrum of clinical manifestations, ranging from asymptomatic cases to severe heart failure or sudden cardiac death. The incidence and prevalence of myocarditis have been observed to vary globally, influenced by factors such as age, sex, and socioeconomic status. Importantly, the elderly are particularly at risk due to age-related changes in the immune system and the presence of comorbidities, which can complicate the clinical picture and management of myocarditis (1–3).

This study aims to analyze the global burden of myocarditis among individuals aged 65 and older from 1990 to 2021, utilizing data from the Global Burden of Disease (GBD) study. We will examine trends in key disease burden indicators such as incidence rates, prevalence, disability-adjusted life years (DALYs), and mortality rates across various regions and populations. Furthermore, we will discuss the implications of our findings for public health policy and clinical practice, particularly in informing strategies for prevention and management of myocarditis in elderly patients (11, 12).

Based on the trends observed in the burden of myocarditis among the elderly population (≥65 years) from 1990 to 2021, variable changes in disease incidence and associated disability-adjusted life years (DALYs) was identified. Although the overall level indicates a downward trend, the risk of disease incidence varies among different countries and regions due to the influence of social economy, living environment, medical level and other factors. Our analysis revealed a nuanced picture. While the age-standardised incidence rate exhibited a slight but significant decline, the absolute number of incident cases and deaths among the elderly increased over the study period. This suggests that the declining individual risk is being offset by the expansion and aging of the global population, leading to a growing absolute burden of myocarditis that requires clinical and public health attention. The longitudinal analysis indicates a significant shift in the age and gender distribution of myocarditis cases, with higher incidence rates observed in older males compared to females. This aligns with earlier findings that highlight the increased susceptibility of male patients to myocarditis, particularly in younger age groups, while women tend to be affected more frequently post-menopause (3).

As the population ages, the burden of myocarditis appears to escalate, necessitating focused public health interventions aimed at prevention and timely management to alleviate the strain on healthcare systems. The data suggest that the elderly population is at heightened risk, with increasing rates of hospitalization and complications associated with myocarditis (2). Furthermore, the association between existing comorbid conditions, such as diabetes and cardiovascular diseases, and the heightened risk of developing myocarditis underscores the importance of comprehensive geriatric assessments in clinical practice (11). Myocarditis exhibits distinct age-specific patterns in its incidence. Prior studies have consistently identified young adults and older adults as high-risk populations for myocarditis. Notably, data from China in 2019 revealed a marked shift toward higher disability-adjusted life years (DALYs) in older age groups, highlighting an emerging trend of aging-related disease burden that warrants greater clinical and public health attention. Myocarditis is an acute and potentially fatal condition; individuals with genetic susceptibility, impaired immunity, or other risk factors may succumb to fulminant myocarditis, heart failure, or arrhythmias earlier in life, thereby contributing to survivorship bias—where only more resilient individuals survive into advanced age. Meanwhile, the elderly population frequently presents with multiple comorbidities, including coronary heart disease, heart failure, cancer, chronic obstructive pulmonary disease (COPD), and renal failure. Although aging is associated with immune dysregulation—potentially increasing vulnerability to infections and exaggerated inflammatory responses—the observed decline in myocarditis burden among the oldest age groups may reflect competing risks of mortality from these comorbid conditions. The etiology of myocarditis remains incompletely understood, with viral infections being the most common cause. Viral infections remain a primary trigger for myocarditis. The recent COVID-19 pandemic has highlighted this link, with SARS-CoV-2 infection itself being a significant cause of acute myocardial injury and myocarditis, particularly in severe cases. In reviewing the temporal trends, it becomes evident that the rise in myocarditis cases may correlate with environmental factors and viral infections, such as those observed during the COVID-19 pandemic (13). However, with the expanding use of immune checkpoint inhibitors in oncology, immune checkpoint inhibitor-associated myocarditis has emerged as an increasingly important contributor to disease burden and requires further investigation.

An interesting finding within our elderly cohort was a decline in myocarditis incidence with advancing age. While this could reflect a true decrease in susceptibility, it is more plausibly explained by epidemiological biases. Survivorship bias is a key factor; individuals who survive into the oldest age groups (e.g., >85 years) are a select group with inherently robust health and potentially different immune responses. Furthermore, competing risks play a major role; in very old patients, acute presentations like myocardial infarction, heart failure, or other infections may be prioritized diagnostically, and myocarditis may be under-investigated or missed. Non-specific symptoms in this frail elderly population may also lead to under-diagnosis. Therefore, the observed age-specific trend should be interpreted with caution, as it likely reflects a combination of diagnostic intensity and competing mortality risks rather than a pure biological phenomenon.

These biological, clinical, and methodological factors—survivorship bias, competing risks, and diagnostic underascertainment due to atypical presentations—may collectively distort observed age trends. To obtain a more accurate understanding of myocarditis epidemiology in older adults, future research should employ longitudinal cohort designs to track changes within the same population over time. Additionally, analytical approaches such as competing risk models should be applied to account for alternative causes of death. Enhanced clinical awareness is essential, particularly regarding the atypical symptom profile of myocarditis in the elderly—including the very old—and clinicians should maintain a high index of suspicion when infectious triggers are present.

As we project into the future, particularly towards 2050, the anticipated increase in the overall burden of myocarditis will likely reflect global demographic changes, including an aging population. Policymakers and healthcare providers must prioritize strategies that address these emerging trends, ensuring that resources are allocated effectively to manage the expected rise in myocarditis cases among the elderly demographic (2). These strategies should include educational initiatives aimed at healthcare professionals and the public, fostering awareness of myocarditis and its potential complications in older adults.

Low-temperature environments have been shown to significantly influence the pathogenesis and prognosis of heart diseases, including coronary heart disease, myocarditis, and heart failure. Multiple studies have demonstrated that exposure to cold temperatures can elevate the risk of cardiovascular events. One primary mechanism involves the activation of the sympathetic nervous system. Cold exposure induces vasoconstriction, thereby increasing blood pressure and cardiac workload. This cascade can lead to endothelial dysfunction and promote atherosclerosis, a key underlying cause of coronary heart disease (14). Beyond the sympathetic nervous system, cold temperatures also impact the immune system. Research indicates that cold stress can suppress immune responses, rendering the body more vulnerable to infections. Such infections, particularly viral myocarditis, can exacerbate heart disease and contribute to poorer outcomes. Low temperatures may suppress immune responses, increase susceptibility to viral infections, and subsequently lead to myocarditis (15). Additionally, low temperatures affect lipid metabolism. Cold exposure has been shown to increase lipid accumulation in adipose tissue and the liver, leading to dyslipidemia. Dyslipidemia is a recognized risk factor for cardiovascular diseases and can accelerate the progression of heart failure (16). The effects of low-temperature environments on heart diseases extend beyond the acute phase, with long-term implications as well. Chronic cold exposure may induce structural and functional changes in the heart, such as left ventricular hypertrophy and diastolic dysfunction, further increasing the risk of heart failure and diminishing overall cardiac function (17). According to our analysis, low-temperature environments may significantly impact the pathogenesis and prognosis of myocarditis. Understanding these mechanisms is essential for developing effective preventive and therapeutic strategies. Future research should explore the complex interactions involved and to develop targeted interventions to minimize the adverse effects of low temperatures on heart health. This correlation emphasizes the need for ongoing surveillance and research to identify potential risk factors and implement effective interventions in at-risk populations. The findings also highlight the disparities in disease burden across different regions, suggesting that socio-demographic factors may influence the prevalence and outcomes of myocarditis among the elderly (14).

The limitations of this research study primarily stem from the inherent challenges associated with global health data collection and the variability in reporting practices across different countries. While we utilized the Global Burden of Disease (GBD) database, which aims to provide comprehensive and standardized data, discrepancies in diagnostic criteria, healthcare access, and resource allocation can significantly affect the accuracy of reported rates for myocarditis. Underreporting in certain regions may result in an underestimation of the global disease burden, particularly in low-sociodemographic index (SDI) areas. Additionally, the potential for underreporting in regions with limited healthcare infrastructure may lead to an underestimation of the true burden of disease. Furthermore, the reliance on estimated annual percentage change (EAPC) for trend analysis may overlook fluctuations in incidence and prevalence that arise from sudden public health events or changes in healthcare policies, thereby limiting the generalizability of our findings.

In conclusion, this study highlights the urgent need for targeted public health interventions to address the growing burden of myocarditis among the elderly population globally. The projected increases in incidence and prevalence underscore the importance of strengthening healthcare systems and enhancing surveillance mechanisms to better capture the complexities of this condition. By understanding the demographic and epidemiological trends, policymakers can formulate evidence-based strategies to mitigate the impact of myocarditis, ultimately improving health outcomes for older adults. Future research should focus on longitudinal studies and multi-center collaborations to refine our understanding of the factors influencing myocarditis burden, ensuring a comprehensive approach to disease prevention and management.

## Data Availability

Publicly available datasets were analyzed in this study. This data can be found here: https://vizhub.healthdata.org/gbd-results/.
